# Interaction Effects of *BDNF* and *COMT* Genes on Resting-State Brain Activity and Working Memory

**DOI:** 10.3389/fnhum.2016.00540

**Published:** 2016-11-02

**Authors:** Wen Chen, Chunhui Chen, Mingrui Xia, Karen Wu, Chuansheng Chen, Qinghua He, Gui Xue, Wenjing Wang, Yong He, Qi Dong

**Affiliations:** ^1^State Key Laboratory of Cognitive Neuroscience and Learning and IDG/McGovern Institute for Brain Research, Beijing Normal UniversityBeijing, China; ^2^Center for Collaboration and Innovation in Brain and Learning Sciences, Beijing Normal UniversityBeijing, China; ^3^Department of Psychology and Social Behavior, University of CaliforniaIrvine, CA, USA; ^4^Faculty of Psychology, Southwest UniversityChongqing, China

**Keywords:** *COMT*, *BDNF*, regional homogeneity, interaction effect, working memory

## Abstract

Catechol-O-methyltransferase (*COMT*) and brain-derived neurotrophic factor (*BDNF*) genes have been found to interactively influence working memory (WM) as well as brain activation during WM tasks. However, whether the two genes have interactive effects on resting-state activities of the brain and whether these spontaneous activations correlate with WM are still unknown. This study included behavioral data from WM tasks and genetic data (*COMT* rs4680 and *BDNF* Val66Met) from 417 healthy Chinese adults and resting-state fMRI data from 298 of them. Significant interactive effects of *BDNF* and *COMT* were found for WM performance as well as for resting-state regional homogeneity (ReHo) in WM-related brain areas, including the left medial frontal gyrus (lMeFG), left superior frontal gyrus (lSFG), right superior and medial frontal gyrus (rSMFG), right medial orbitofrontal gyrus (rMOFG), right middle frontal gyrus (rMFG), precuneus, bilateral superior temporal gyrus, left superior occipital gyrus, right middle occipital gyrus, and right inferior parietal lobule. Simple effects analyses showed that compared to other genotypes, subjects with *COMT-*VV*/BDNF-*VV had higher WM and lower ReHo in all five frontal brain areas. The results supported the hypothesis that *COMT* and *BDNF* polymorphisms influence WM performance and spontaneous brain activity (i.e., ReHo).

## Introduction

Working memory (WM) refers to a limited capacity system allowing the temporary storage and manipulation of information (Baddeley and Hitch, 1974) and plays a crucial role in a wide range of cognitive tasks such as reading, problem solving, and learning (Baddeley and Della Sala, 1983; Conway et al., 2003). Researchers have recently examined the genetic underpinnings of WM and identified two candidate genes, Catechol-O-methyltransferase (*COMT*) and brain-derived neurotrophic factor (*BDNF*; Egan et al., 2001; Numata et al., 2007; Nagel et al., 2008; Wang et al., 2013).

*COMT* has been found to be associated with dopamine concentration in the prefrontal cortex (PFC; Lachman et al., 1996b; Akil et al., 2003; Chen et al., 2004), which is in turn associated with WM (Egan et al., 2001). The *COMT* Met allele has also been found to be associated with attention shifting, which plays an important role in the updating of WM (Nolan et al., 2004) and efficient physiological responses during the two-back WM task (Egan et al., 2001). Interestingly, studies have found ethnic differences in the effects of *COMT*. Most studies that used Caucasian samples (Bruder et al., 2005; Aguilera et al., 2008) found that compared with the Val allele, the Met allele was correlated with better cognitive abilities, especially WM. On the other hand, in Asian samples, the Met allele was instead associated with higher incidence of major depressive disorder (MDD; Wang et al., 2016), lower IQ (Qian et al., 2009), lower scores on competency tests (Yeh et al., 2009), and lower WM (Wang et al., 2013).

*BDNF* plays an important role in neuronal survival and differentiation and synaptic plasticity. Even though not all studies are consistent (for exceptions, see Egan et al., 2003; Hansell et al., 2007), there is a growing number of studies showing associations between *BDNF* (and its product BDNF) and cognitive ability. On the biological level, several animal studies have shown that performance on WM tasks can be affected by both BDNF overexpression (Papaleo et al., 2011) and under-expression in mice (Li et al., 2010). Higher BDNF levels in the frontal cortex have been linked to fewer WM errors in aged rats (Bimonte et al., 2003) and Ts65D mice (Bimonte-Nelson et al., 2003). In human studies, *BDNF* rs6265 Val/Met polymorphisms have been correlated with WM. A study of healthy Han Chinese found that *BDNF* rs6265 Val homozygotes performed better in digital WM tasks than did Met homozygotes (Gong et al., 2009). In studies of bipolar patients, the Met variant of *BDNF* (rs6265) has been associated with altered hippocampus and PFC formation, which are in turn linked to poorer WM performance. Egan et al. (2003) found that a common *BDNF* (val66met) polymorphism was associated with episodic memory, although not with WM as mentioned earlier.

It should be mentioned that due to genetic pleiotropy, both *COMT* and *BDNF* have been found to be associated with several other aspects of cognition beyond WM, including reaction time (Das et al., 2014), cognitive flexibility (Kang et al., 2013), aging-related cognitive degradation (Ghisletta et al., 2014; Voelcker-Rehage et al., 2015), executive function (Leckie et al., 2014; Sapkota et al., 2015), and neurocognitive deficits in people with schizophrenia (Ahmed et al., 2015) or Parkinson's disease (Khalil et al., 2016). Nevertheless, WM has been the main focus of the previous literature in this field.

In terms of specific interaction effects of the two genes on WM, however, there was only one study that found that the effect of *COMT* on WM was modulated by *BDNF* in a sample of aging Germans (Nagel et al., 2008). Other studies suggested such an effect indirectly by demonstrating similar interaction effects on the anatomy of the hippocampus (Hajek et al., 2012) and PFC (Gothelf et al., 2005; Ho et al., 2007; Takahashi et al., 2008), brain cortical plasticity and frontostriatal circuit brain activities (Wang et al., 2015), all of which could affect memory performance. Interactions between *COMT* and *BDNF* polymorphisms exist for WM likely because both genes are widely expressed in the cortex and white matter (in humans and mice, see The Human Protein Atlas, http://www.proteinatlas.org/, and Allen Brain Atlas, http://www.brainatlas.org) (Su et al., 2004). They both have been found to influence dopamine levels in the midbrain (Meyer-Lindenberg et al., 2005; Berton et al., 2006) and the PFC (Lachman et al., 1996a; Gogos et al., 1998; Malhotra et al., 2002; Chen et al., 2004).

The current study aimed to explore the potential interactions between *BDNF* Val66Met and *COMT* Val158Met on resting brain regional homogeneity (ReHo) as well as WM in a sample of Chinese subjects. Resting-state fMRI has increasingly been used to investigate neural basis of cognitive ability including WM (Christoff and Gabrieli, 2000; Stark and Squire, 2001) both in patients and in healthy people. For example, a previous study found that resting-state functional connectivity was positively correlated with WM performance (Hampson et al., 2006). Zou et al. (2013) further found that the amplitude of low-frequency fluctuation (ALFF) of certain brain areas such as the precuneus was correlated with WM performance (Zou et al., 2013). ReHo, calculated by using Kendall's coefficient concordance (KCC) to measure the degree of synchronization between the time-series of a voxel and its nearest voxels, is believed to reflect the regional spontaneous blood oxygenation level dependent (BOLD) fluctuations of the whole brain during rest (Zang et al., 2004). Many studies have found abnormal ReHo associated with various brain diseases, such as depression (Guo et al., 2011; Peng et al., 2011), Parkinson's disease (Wu et al., 2009), autism spectrum disorder (Paakki et al., 2010), and Alzheimer's disease and mild cognitive impairment (Zhang et al., 2012). Compared to resting-state functional connectivity (RSFC), which focuses on the long-distance interregional temporal correlations of BOLD signals, ReHo focuses on the functions within each region, in the case of this study, the frontal lobe. Although, no study has examined the effects of *COMT* and *BDNF* on ReHo of resting-state brain activity, the aforementioned literature review led us to hypothesize that *COMT* and *BDNF* would have main and interactive effects on both WM and ReHo.

## Experimental procedures

### Participants

Participants were Chinese college students recruited from Beijing Normal University. Genetic data (*BDNF* and *COMT*) and WM data were available for 428 participants, but 11 participants were excluded from further analysis because they were outliers in their WM performance (outside of 3 standard deviations of the group mean). Of the remaining 417 subjects (57% female, mean age = 21.31 years), resting state fMRI data were available for 298 participants (Table 1). All subjects were Han Chinese and reported no history of psychiatric diseases, head injuries, or stroke/seizure. Moreover, scores on the Beck Depression Inventory (Beck et al., 1996) and Beck Anxiety Inventory (Beck and Steer, 1990) showed that none of the participants met the criteria for either major depression or anxiety disorder. No participants met the criterion for nicotine dependence according to the Fagerström Test for Nicotine Dependence (Heatherton et al., 1991), but four participants in the larger sample reported using a large amount of alcohol based on the Alcohol Use Disorders Identification Test (Saunders et al., 1993). This experiment was approved by the Institutional Review Board (IRB) of the State Key Laboratory of Cognitive Neuroscience and Learning at Beijing Normal University, China. A written consent form was obtained from each participant after a full explanation of the study procedure. Participants were compensated for their participation.

**Table 1:** **Demographic information for *COMT* and *BDNF* genotype groups**.

**Variables**	***COMT-VV***			***COMT-VM***			***COMT-MM***			***F***	***P***
	***BDNF-VV***	***BDNF-VM***	***BDNF-MM***	***BDNF-VV***	***BDNF-VM***	***BDNF-MM***	***BDNF-VV***	***BDNF-VM***	***BDNF-MM***		
**SUBJECTS WITH DATA ON THE WM TASK (*****N*** = **417)**											
Group size (M/F)	61 (28/33)	109 (49/60)	67 (34/33)	43 (18/25)	82 (28/54)	32 (15/17)	9 (2/7)	10 (3/7)	4 (2/2)	See note	
Age (mean ±*SD* in years)	21.18 ± 0.87	21.39 ± 0.84	21.40 ± 1.06	21.39 ± 0.84	21.23 ± 0.72	21.14 ± 0.90	21.44 ± 0.75	21.06 ± 0.79	22.32 ± 0.78	1.52	0.15
IQ (mean ±*SD*)	125.73 ± 7.81	126.47 ± 7.80	127.17 ± 7.69	124.79 ± 9.12	124.90 ± 7.71	124.52 ± 8.42	125.38 ± 8.15	129.93 ± 8.23	128.50 ± 7.37	0.94	0.48
**SUBJECTS WITH DATA FROM fMRI (*****N*** = **298)**											
Group size (M/F)	41 (13/28)	77 (28/49)	46 (18/28)	29 (8/21)	62 (14/48)	23 (9/14)	8 (1/7)	8 (2/6)	4 (2/2)	See note	
Age (mean ±*SD* in years)	21.09 ± 0.14	21.41 ± 0.10	21.34 ± 0.13	21.34 ± 0.16	21.17 ± 0.11	21.11 ± 0.18	21.38 ± 0.31	20.87 ± 0.31	22.32 ± 0.43	1.74	0.09
IQ (mean ±*SD*)	126.10 ± 7.87	126.16 ± 7.85	127.50 ± 7.54	126.86 ± 8.75	124.70 ± 7.40	124.59 ± 7.29	125.38 ± 7.98	123.87 ± 11.28	128.00 ± 8.16	0.64	0.74

Note:“VV” stands for the Val homozygotes, “VM” stands for the Val/Met heterozygotes, “MM” stands for the Met homozygotes, “M/F” stands for the numbers of males and females, “SD” stands for standard deviation, and “WM” stands for working memory. Genotype distributions did not differ by gender: COMT (χ^2^ = 4.01, p = 0.14 in the 417 sample, and χ^2^ = 2.86, p = 0.24 in the 298 sample) and BDNF (χ^2^ = 2.64, p = 0.27 in the 417 sample, and χ^2^ = 2.81, p = 0.24 in the 298 sample).

### Material and cognitive tasks

WM performance was assessed by a two-back WM paradigm with three subtasks (semantic, phonological, and morphological judgment). Each task included four blocks of 10 trials each. Participants were asked to make continuous judgments between the word currently presented and the word presented two trials earlier in terms of their semantic (whether they belonged to the same semantic category), phonological (whether they rhymed with each other), or morphological relations (whether they were the same Tibetan character; Xue et al., 2004). For more details on the specific procedure, please see Li et al. (2011). Following the previous studies (Fry and Hale, 1996; Rosenheck et al., 2006; Zhu et al., 2010; Li et al., 2011; Wang and Gathercole, 2013), the average accuracy scores of the three WM subtasks were used in order to obtain a more stable and reliable estimate of WM. We also measured the participants' IQ with the Wechsler Adult Intelligence Scale. IQ was used as a control variable.

### Genotyping

The genomic DNA for each participant was extracted from a 4 ml venous blood sample and purified by standard procedures. The *COMT* val158met was genotyped using PCR (Qian et al., 2003) with the following primers (forward: 5′-TCGTGGACGCCGTGATTCAGG-3′ and reverse: 5′-ACAACGGGTCAGGCATGCA-3′). The PCR mixture consisted of × 1 Taq Buffer (MBI), 0.2 mM dNTP, 0.25 mM primers, 1 mM MgCl2, 1.0 unit Taq polymorphism and 100 ng DNA. PCR was initiated at 95⋅C for 3 min, then at 94⋅C for 30 s, 68⋅C for 60 s, 72⋅C for 90 s for 35 cycles, then extended at 72⋅C for 7 min. The Val158Met variation was differentiated using NlaIII restriction fragment length polymorphism analysis on 4% agarose gel electrophoresis. Two common fragments (81 and 22 bp) and variable fragments of 114 bp (Val158/wild type) and 96 + 18 bp (Met158/mutant) were available. Genotypes were determined from Gel Doc 2000 (Bio-Rad, Hercules, CA). Because the short fragments (22 and 18 bp) diffused in the agarose gel, only three bands (114, 96, and 81 bp) were detectable.

*BDNF* was genotyped through the polymerase chain reaction-restriction fragment length polymorphism (PCR-RFLP) method. The forward primer was ITS-1: (5′-ACTCTGGAGAGCGTGAAT-3′) and the reverse primer was ITS-2: (5′-ATACTGTCACACACGCTC-3′). The PCR amplification reactions were performed in a 15 μl reaction mixture containing 2 μl template DNA, 1.5 μl MgCl2, 0.3 μl of each primer, 0.3 μl dNTP, 0.12 μl Taq polymerase, 1.5 μl 10 × buffer, 3 μl PCR Optimizer, and water up to a total volume of 15 μl. The thermocycler conditions were as follows: 95⋅C for 5 min, 35 cycles of 95⋅C for 30 s, 62⋅C for 30 s, 72⋅C for 30 s, and a final extension at 72⋅C for 10 min. Amplified products of 308 bp was electrophoresed in a 2% agarose gel and visualized with ethidium bromide. The polymorphism was genotyped via restriction digestion with 5 μl of the PCR product, 2U Eco72I, 0.8 μl buffer, 0.08 μl BSA, and water up to the total volume of 8 μl. The digested products were electrophoresed on a 2% agarose gel and visualized with ethidium bromide staining.

Ambiguous or unidentifiable results were re-amplified and rescored. A subset of the samples (10%) were randomly selected and tested twice for confirmation. The detection rates of *COMT* and *BDNF* were 97.9 and 99.5%, respectively, in the original larger sample, but only subjects with good genotype data were later enrolled in the WM and brain imaging study.

### Brain imaging data collection and preprocessing

#### fMRI data acquisition

MR images were collected using a SIEMENS TRIO 3-Tesla scanner in the Brain Imaging Center of Beijing Normal University. Participants lay supine with their heads snugly fixed by a belt and foam pads to minimize head motion. Each participant underwent an 8 min resting state fMRI (RS-fMRI) scanning session and a 3D anatomic session. During the RS-fMRI session, the participants were instructed to keep their eyes closed, be as still as possible, and not to think about anything in particular. Images were obtained with the following parameters: 33 axial slices, thickness/gap = 3/0.6 mm, matrix = 64^*^64, repetition time (TR) = 2000 ms, echo time (TE) = 30 ms, flip angle = 90⋅, field of view (FOV) = 200^*^200 mm^2^. The 3D T1-weighted magnetization-prepared rapid gradient echo (MPRAGE) image was acquired with the following parameters: 128 sagittal slices, slice thickness/gap = 1.33/0 mm, matrix = 256^*^256, TR = 2530 ms, TE = 3.39 ms, inversion time (Ti) = 1100 ms, flip angle = 7⋅, FOV = 256^*^256 mm^2^.

#### Image preprocessing

Data Processing Assistant for Resting-State fMRI (DPARSF, http://rfmri.org/DPARSF; Yan and Zang, 2010) was used to preprocess the RS-fMRI data. Steps included: (1) discarding the first 10 volumes; (2) correcting for within-scan acquisition time differences between slices and head motions; (3) coregistering the T1 image to the mean functional image using a linear transformation; (4) segmenting the coregistered T1 images into gray matter, white matter and cerebrospinal fluid; (5) normalizing the head motion corrected functional images to standard template using the transformation matrix estimated from T1 segmentation and reslicing them to 3 mm isotropic resolution; (6) linear detrending and temporal band-pass filtering (0.01~0.08 Hz) to reduce the effects of low-frequency drift and high-frequency physiological noise; and (7) regressing out the nuisance signals including the six head motion profiles, global mean signal, cerebrospinal fluid signal, and white matter signal. ReHo was calculated from these preprocessed images using Kendall coefficient of concordance (KCC; Kendall and Gibbons, 1990) based on 27 nearest neighboring voxels. Then the ReHo maps were spatially smoothed (FWHM = 6 mm) and standardized by dividing the maps by the mean ReHo of the entire brain (Zang et al., 2004).

### Data analysis

ANCOVA was used to examine the effects of *COMT* and *BDNF* on WM performance with gender, age, and IQ as covariates. With the larger (*N* = 417) sample with genetic and WM data, we ran a *COMT*(3 groups) ^*^ *BDNF*(3 groups) ANCOVA.

Genetic effects on resting brain state were analyzed using SPM8 (http://www.fil.ion.ucl.ac.uk/spm) implemented in Matlab 7.8 (The Mathworks, Massachusetts, US) for Windows. Considering the smaller sample size with imaging data, we followed previous research (Li et al.'s, 2016) and combined M/V with M/M to form the Met-carrier group. Subjects were divided into four groups, ordered from high to low in presumed dopamine signaling (see Li et al.'s, 2016): *COMT*-M+/*BDNF*-VV, *COMT*-M+/*BDNF*-M+, *COMT*-VV/*BDNF*-VV, and *COMT*-VV/*BDNF*-M+. The design matrix was specified with four regressors defined as “1” (subject in the group) or “0” (subject not in the group). A random effects general linear model was fitted onto ReHo maps with age, gender, and IQ as covariates. Genetic main and interaction effects maps were obtained by setting up contrasts, and Region of Interest (ROI) analysis was further conducted to explore interactions by defining the significant clusters located in the frontal lobe as ROIs and averaging the ReHo in each ROI for each subject. We focused on the frontal lobe for two main reasons. First, the frontal lobe has been consistently found to be a vital brain area correlated with WM in lesion patients and healthy populations (Owen et al., 1990; Curtis and D'Esposito, 2003; Barbey et al., 2013). Most neuroimaging studies have targeted the frontal cortex to investigate the neural basis of N-back WM (Brunoni and Vanderhasselt, 2014). Second, despite the widely distributed expression of *COMT* and *BDNF* gene in the brain, studies have found that WM was associated with dopamine concentration in the PFC (Lachman et al., 1996b; Akil et al., 2003; Chen et al., 2004) and was affected by *COMT* and *BDNF* levels in the frontal cortex (Bimonte et al., 2003). Then WM was correlated with the averaged ReHo in each ROI. We also regressed WM to whole-brain ReHo to confirm the relationship between ReHo and WM. Furthermore, as the global brain signal regression (GSReg) may lead to artificial results (Gotts et al., 2013; Saad et al., 2013), we reanalyzed our data without GSReg to confirm the stability of the findings.

For this study, the significance threshold was set at 0.05 for the omnibus *F*-test in ANCOVA. When the omnibus *F*-value was significant, it was followed by LDS *post-hoc* test, *p* < 0.05. The statistical results of the whole-brain analysis of fMRI data were corrected for multiple comparisons using the “AlphaSim” implemented in REST (http://www.restfmri.net; Song et al., 2011). This function is based on the Monte Carlo simulation in AFNI (https://afni.nimh.nih.gov/pub/dist/doc/manual/AlphaSim.pdf). Voxels with *p* < 0.01 and cluster Size > 459 mm^3^ (17 voxels) were considered significant, which corresponds to corrected *p* < 0.05.

## Results

The mean IQ of the sample was 125.92 for the 417 sample and 125.94 for the 298 sample.

### Genotype distribution

For the *COMT* gene, 237 subjects were V/V, 157 were V/M, and 23 were M/M. The sample was in Hardy–Weinberg Equilibrium (HWE, χ^2^ = 0.11, *p* > 0.05). The Met minor allele frequency in our sample was 0.24, which is comparable to the HapMap data for Han Chinese (0.29 for Han Chinese in Beijing and 0.26 for Chinese in Metropolitan, Denver, Colorado, according to the HapMap Genome Browser release #28).

For the *BDNF* gene, the genotype frequencies (113 V/V, 201 V/M, and 103 M/M) were also in HWE (χ^2^ = 0.31, *p* > 0.05). The frequency of the *BDNF* Met allele (0.49) in our sample is similar to other Asian data, 0.41 in a Japanese sample (Shimizu et al., 2004), 0.47 in a Chinese Han population (Yi et al., 2011), and 0.47 in a Korean sample (Kim et al., 2007).

The distribution of genotypes did not vary significantly by gender, age, and IQ for either the total 417 sample or the 298 subsample (Table 1). They were nevertheless used as covariates in the main analyses.

### Genetic polymorphisms and WM

For the whole sample (*N* = 417), the mean accuracy on the WM task was 0.86 (*SD* = 0.06). Males and females had comparable mean accuracy, 0.85 (*SD* = 0.06) and 0.86 (*SD* = 0.06), respectively, *F*_(1, 415)_ = 1.27, *p* = 0.26.

**Figure 1:**
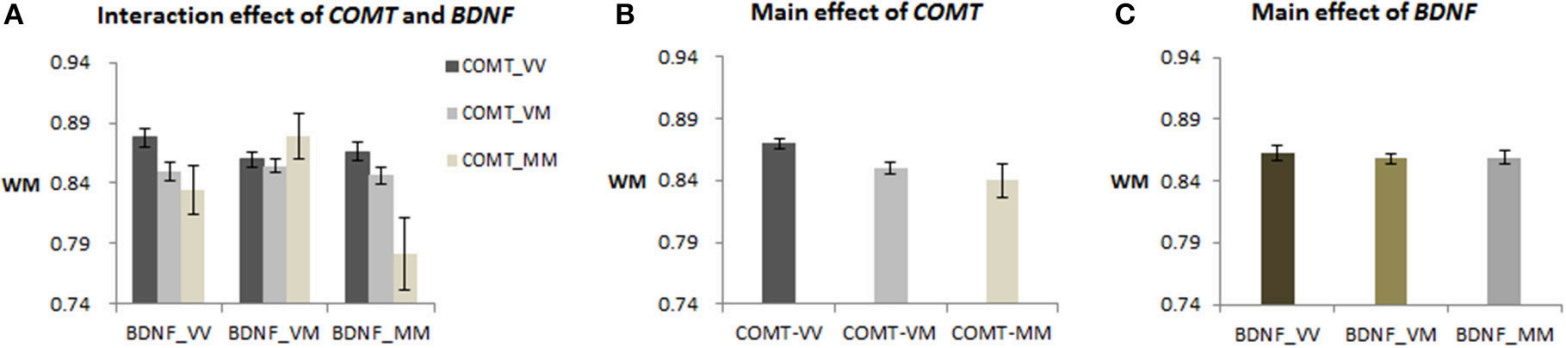
***COMT* and *BDNF* gene effect on WM performance in the 417 sample. (A)** The interaction effect of *COMT* and *BDNF* (*post-hoc* LSD test showed that *COMT*-VV was higher than *COMT*-MM for both *BDNF*-VV and *BDNF*-MM groups, *p* < 0.05); **(B)** The main effect of *COMT* (*post-hoc* LSD test showed that *COMT*-VV was higher than *COMT*-VM, *p* < 0.05); **(C)** The main effect of *BDNF* (*post-hoc* LSD test showed no significant contrasts).

A 3 × 3 genotypes ANCOVA demonstrated that both genes had significant main effects on WM [*COMT*: *F*_(2, 405)_ = 5.92, *p* = 0.003; *BDNF*: *F*_(2, 405)_ = 3.09, *p* = 0.046], and the interaction effect also was significant [*F*_(4, 405)_ = 2.62, *p* = 0.034] in the whole sample, *N* = 417 (see Figure 1A). *Post-hoc* analysis of the *COMT* main effect showed that Val homozygotes had higher WM (*M* = 0.867) than the heterozygotes (*M* = 0.851), *p* = 0.033, but neither group differed from the Met homozygotes (*M* = 0.845) (see Figure 1B), partly due to the latter group's small sample size. *Post-hoc* analyses of the *BDNF* main effect did not reveal significant contrasts (see Figure 1C). Finally, the interaction effect of the two genes was due to significant simple effects of *COMT* for the two groups of individuals who were homozygous for *BDNF* (i.e., V/V and M/M, *p* = 0.029 and 0.016, respectively), but not for the *BDNF*-VM individuals. After combining the VM and MM, the *COMT*-VV was still higher than *COMT*-M+ for *BDNF*-VV [*F*_(1, 410)_ = 8.42, *p* = 0.004; see Figure S1].

### Genetic polymorphisms and ReHo

**Figure 2:**
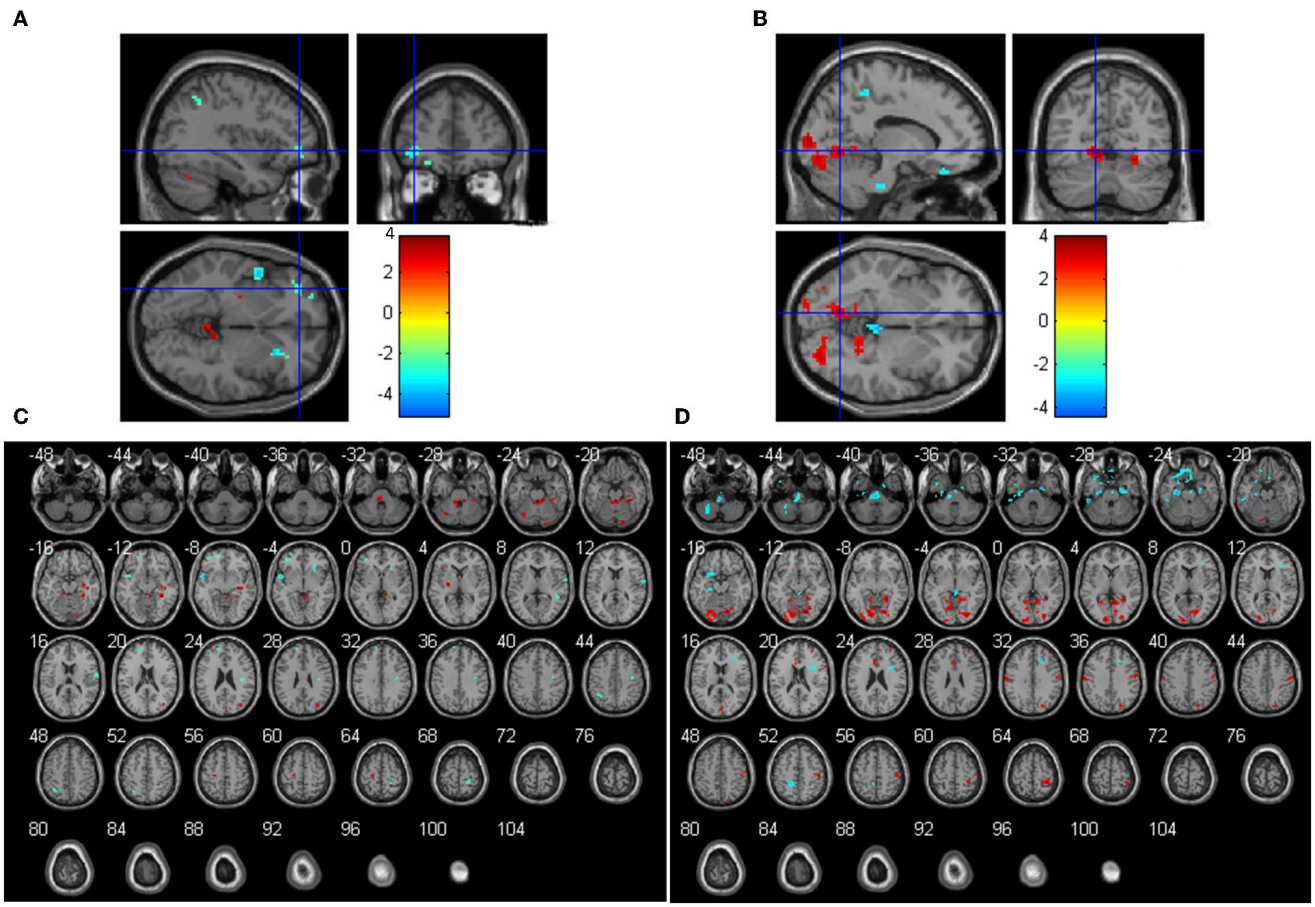
**Main effects of COMT (A,C) and BDNF (B,D) on ReHo (*N* = 298)**. The coordinates of the cross in **(A)** and **(B)** are [−36, 45, −3] and [−12, −66, −3], respectively. Positive *t*-values mean that the Val homozygotes showed higher ReHo than the Met carriers and negative t values mean that the Val homozygotes showed lower ReHo than the Met carriers.

The main effects of the *COMT* gene on ReHo were significant in several regions. *COMT* Val homozygotes had significant higher ReHo than the *COMT* Met carriers in the bilateral cerebellum anterior lobe, left cerebellum posterior lobe, left putamen, right hippocampus, fusiform gyrus, and middle occipital gyrus, but lower ReHo in right superior and inferior frontal gyrus, left middle frontal gyrus, bilateral superior temporal gyrus, right postcentral gyrus, bilateral precentral gyrus (Figures 2A,C and Table S1). In terms of the main effects of the *BDNF* gene, Val homozygotes had significant higher ReHo than the *BDNF* Met carriers in the bilateral lingual gyrus, left limbic lobe, right precentral and postcentral gyrus, right precuneus, right superior frontal gyrus, but lower ReHo in left middle and inferior temporal gyrus, right superior and medial frontal gyrus and left inferior frontal gyrus, bilateral parahippocampal gyrus, left paracentral lobule, and bilateral cerebellum posterior lobe (Figures 2B,D and Table S2).

**Figure 3:**
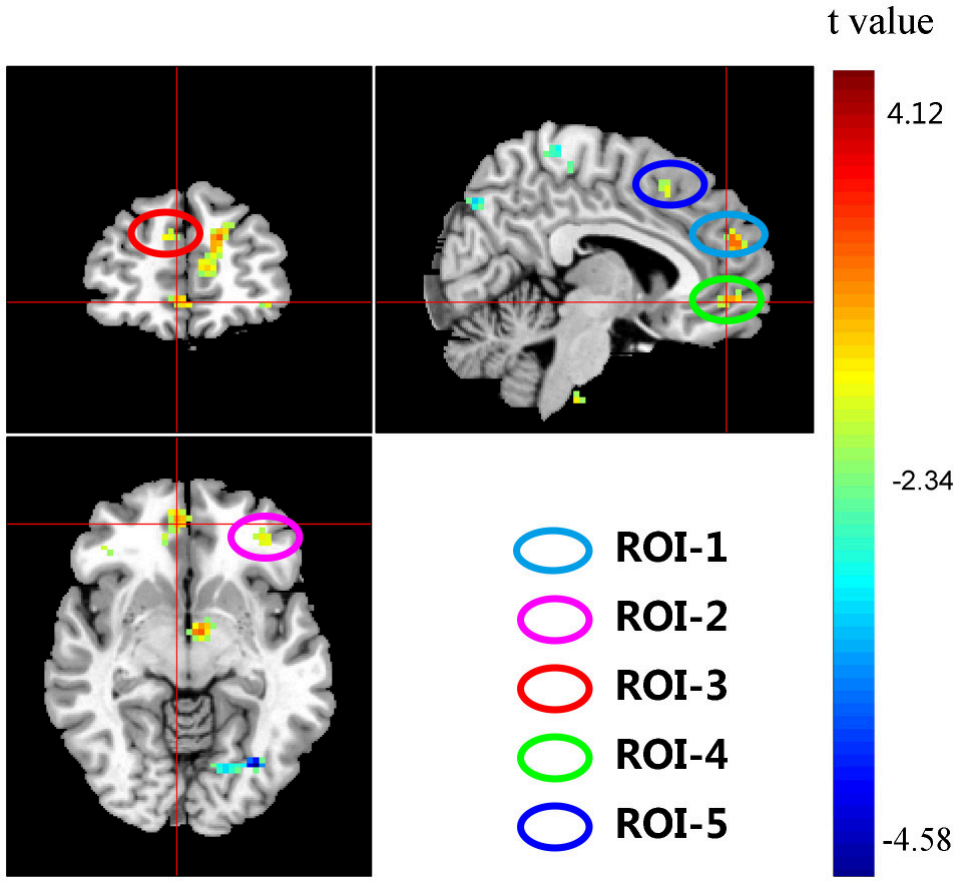
**Interaction effects of *COMT* and *BDNF* on ReHo of the whole brain (*N* = 298)**. The coordinates of the cross are [6, 48, −7]. Circles indicate the five regions of interest (ROIs) located in the frontal cortex: ROI-1, right medial frontal gyrus (rMeFG); ROI-2, left superior frontal gyrus (lSFG); ROI-3, right superior and medial frontal gyrus (rSMFG); ROI-4, right medial orbitofrontal gyrus (rMOFG); and ROI-5, right middle frontal gyrus (rMFG).

Significant interactions between *COMT* and *BDNF* were found in left medial frontal gyrus (lMeFG), left superior frontal gyrus (lSFG), right superior and medial frontal gyrus (rSMFG), right medial orbitofrontal gyrus (rMOFG), right middle frontal gyrus (rMFG), precuneus, bilateral superior temporal gyrus, left superior occipital gyrus, right middle occipital gyrus, and right inferior parietal lobule (Tables 2, 3 and Figure 3).

**Table 2:** **Brain areas showing significant *COMT* and *BDNF* gene interaction effects on ReHo with age, gender, and IQ as covariates, with AlphaSim correction (*p* < 0.01; *N* = 298)**.

**Brain areas**	**Cluster size (Voxels)**	**Brodmann areas (BA)**	**Peak coordinates in MNI**			***T*-value**	***p*-value (uncorrected)**	**ROI**
			**x**	**y**	**z**			
Left medial frontal gyrus(rMeFG)	85	9/10	−9	54	12	3.84	0.000	1
left superior frontal gyrus(lSFG)	46	47	−30	42	6	3.61	0.000	2
			−39	45	−9	3.40	0.000	
			−30	42	−3	3.28	0.001	
Right superior and medial frontal gyrus(rSMFG)	40	9/10	6	51	21	3.53	0.000	3
			12	45	27	3.01	0.001	
Right medial orbitofrontal gyrus(rMOFG)	25	10/11	6	51	−6	3.30	0.001	4
			12	42	−6	2.63	0.004	
Right middle frontal gyrus(rMFG)	24	6	48	0	48	3.25	0.001	5
			36	6	48	3.03	0.001	
Right paracentral lobule	64	4/5/6	−3	−24	63	3.02	0.001	
			6	−36	69	2.79	0.003	
			3	−30	57	2.66	0.004	
Right middle frontal gyrus(rMFG)	54		24	27	9	3.64	0.000	
			27	33	0	3.33	0.000	
			45	39	0	2.66	0.004	
Left insula	47		−27	−27	24	3.90	0.000	
			−30	−36	33	3.24	0.001	
Right middle occipital gyrus(rMOG)	39	19/39	33	−75	24	3.82	0.000	
Left superior occipital gyrus(SOG)	38	18/19	−12	−84	21	3.25	0.001	
			−18	−87	27	2.78	0.003	
Right corpus callosum	38		30	15	27	3.58	0.000	
			12	15	24	2.92	0.002	
			21	12	27	2.72	0.003	
Lingual gyrus	36	18/19	−33	−72	−6	4.12	0.000	
			−18	−72	−6	2.95	0.002	
Right paraHippocampal gyrus	34	28/35	18	−24	−24	3.97	0.000	
Right superior motor areas	30	6/8/32	15	9	57	3.38	0.000	
			9	18	45	3.13	0.001	
Left superior temporal gyrus(lSTG)	28		−42	−33	3	3.59	0.000	
			−36	−39	9	2.68	0.004	
Right precentral gyrus	27		21	−6	51	3.60	0.000	
			36	0	33	3.24	0.001	
Left lentiform nucleus	26	10	−6	−3	−3	4.58	0.000	
Undefined	24		3	−27	−57	3.08	0.001	
Right superior temporal gyrus(lSTG)	22	39	45	−54	18	3.76	0.000	
Right inferior parietal lobule	21	40	45	−36	51	2.77	0.003	
			45	−33	36	2.70	0.004	
Right parahippocampal gyrus	20	28	12	18	−30	2.99	0.002	
			15	3	−33	2.45	0.007	
Right precuneus	20	7	3	−78	42	3.71	0.000	
Left postcentral gyrus	20	4/6	−54	−6	48	3.29	0.001	
Cerebellum anterior lobe	18		30	−48	−24	3.80	0.000	
Left corpus callosum	18		−3	21	3	3.98	0.000	
Undefined	17		−48	−12	−45	3.42	0.000	
			−42	−6	51	3.08	0.001	

**Table 3:** **Main and interactive effects of *COMT* and *BDNF* on mean ReHo in five significant ROIs located in frontal lobe (*N* = 298)**.

**ROI**	**Brain areas**	***F***			***P***		
		***COMT***	***BDNF***	COMT^*^BDNF	***COMT***	***BDNF***	COMT^*^BDNF
1	lMeFG	7.266	2.231	28.011	**0.007**	0.136	**0.000**
2	lSFG	12.568	0.608	25.899	**0.000**	0.436	**0.000**
3	rSMFG	5.011	0.055	16.907	**0.026**	0.814	**0.000**
4	rMOFG	5.917	0.350	13.697	**0.016**	0.554	**0.000**
5	rMFG	1.464	0.007	17.118	0.227	0.934	**0.000**

lMeFG, left medial frontal gyrus; lSFG, left superior frontal gyrus; rSMFG, right superior and medial frontal gyrus; rMOFG, right medial orbitofrontal gyrus; rMFG, right middle frontal gyrus. Significant p-values (< 0.05) are shown in bold.

**Figure 4:**
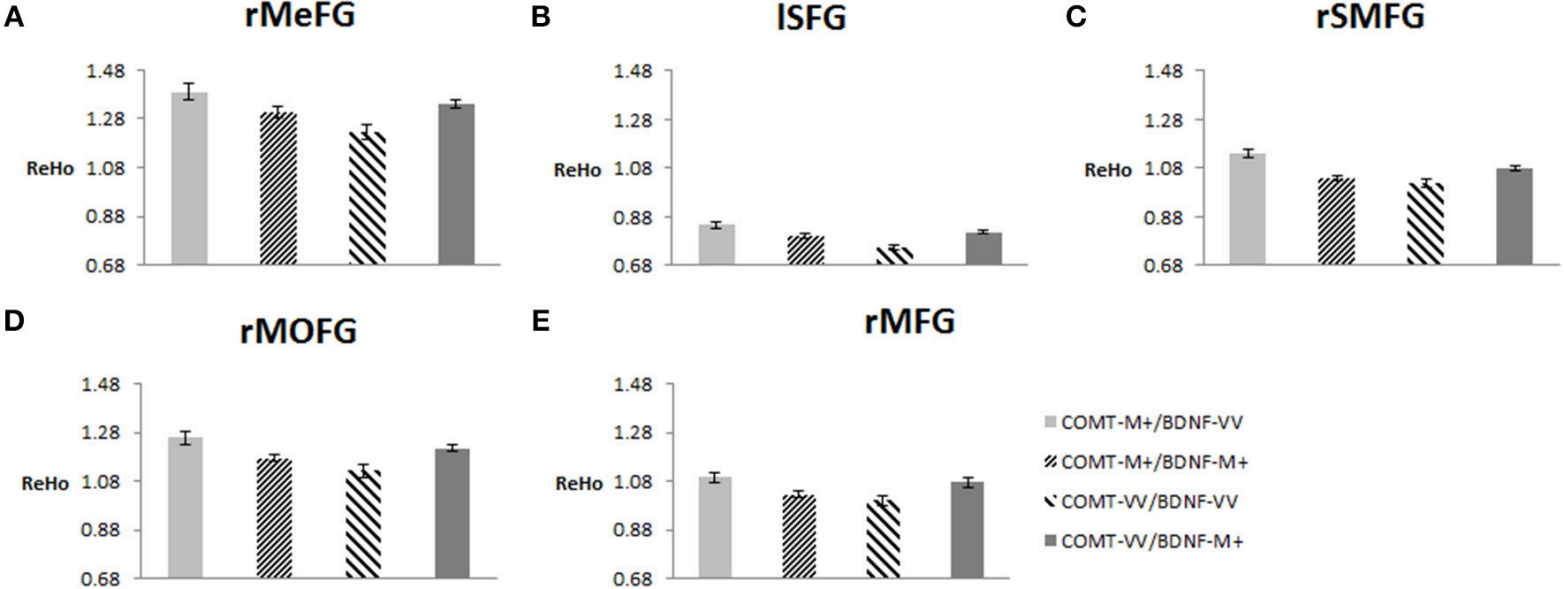
**Effects of COMT and BDNF on mean ReHo of each ROI (*N* = 298). (A)** right medial frontal gyrus (rMeFG); **(B)** left superior frontal gyrus (lSFG); **(C)** right superior and medial frontal gyrus (rSMFG); **(D)** right medial orbitofrontal gyrus (rMOFG); **(E)** right middle frontal gyrus (rMFG). For all five regions of interest (ROIs), each of the middle two groups (i.e., with presumed medium level of dopamine signaling) showed significantly lower ReHo than each of the other two groups (i.e., low or high level of dopamine signaling; *p* < 0.05). Details on additional significant contrasts are available from the authors.

To plot the interaction effects, we focused on the five clusters located in the frontal lobe. The mean ReHo for each ROI was extracted for each subject. Simple effects analyses showed that all five ROIs consistently showed a U-shaped pattern across the four groups arranged along the presumed strength of dopamine signaling. *COMT*-M+/*BDNF*-VV and *COMT*-VV/*BDNF*-M+ had significantly higher ReHo than *COMT*-M+/*BDNF*-M+ and *COMT*-VV/*BDNF*-VV; Figure 4).

**Figure 5:**
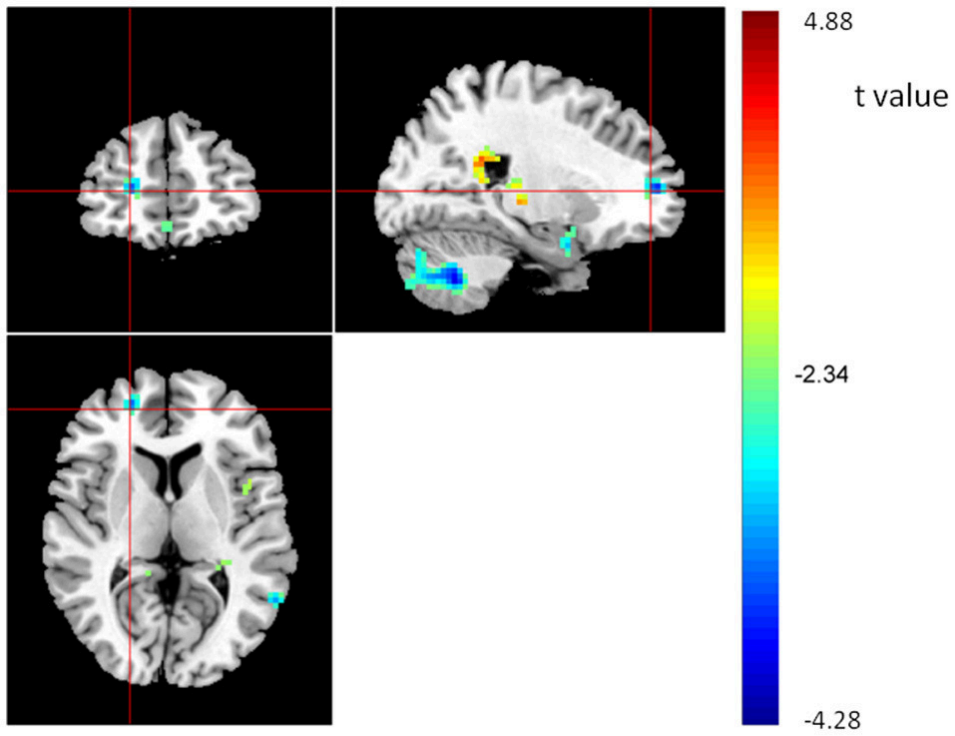
**Brain areas showing positive (red) or negative (blue) correlations with WM, based on whole brain ReHo analysis, with AlphaSim correction (*p* < 0.01; *N* = 298)**. The coordinates of the cross are [22, 50, 7].

Finally, we correlated the average ReHo of each of the five ROIs with WM performance. Results showed that ReHo of rSMFG had a modest negative correlation with WM performance (*r* = −0.122, *p* = 0.036). Moreover, the regression analysis with whole-brain images showed that the ReHo of rSMFG showed a negative correlation with WM performance (see Figure 5 and Table 4). The results of the reanalysis without GSReg showed that, although the number of clusters showing significant *COMT* and *BDNF* gene effect on ReHo decreased from 26 to 19, the rSMFG was still included (Table S3) and showed the same pattern of results as with GSReg (Figure S2). Furthermore, we regressed WM to whole-brain ReHo and confirmed the negative relationship between ReHo and WM (Table S4).

**Table 4:** **Brain areas showing significant correlations between WM and ReHo throughout the whole brain, with AlphaSim correction (*p* < 0.01; *N* = 298)**.

**Brain areas**	**Cluster size (Voxels)**	**Brodmann areas (BA)**	**Peak coordinates in MNI**			***T*-value**	***p*-value (uncorrected)**
			**x**	**y**	**z**		
**BRAIN AREAS SHOWING POSITIVE RELATIONSHIP BETWEEN WM AND ReHo**							
Left superior occipital gyrus	23	7/18/19	−12	−84	24	4.28	0.000
Left precuneus and posterior cingulate gyrus	101	29	−15	−51	21	4.17	0.000
			−18	−36	27	3.28	0.001
			−6	−42	9	2.76	0.003
Right anterior cerebellum lobe	122		3	−54	0	4.08	0.000
			−9	−30	−15	3.35	0.000
			0	−66	−12	3.2	0.001
Left supramarginal gyrus	22	40	−48	−45	36	3.78	0.000
Right cingulate gyrus	97		3	9	15	3.73	0.000
			9	−3	21	3.32	0.001
			−15	3	24	2.92	0.002
Right precuneus	151		18	−45	21	3.63	0.000
			12	−42	12	3.29	0.001
			24	−39	24	3.08	0.001
Right middle cingulum	17	24/32	12	6	33	3.5	0.000
			0	9	42	2.61	0.005
Left cingulate gyrus	19	24/31	−6	0	60	3.37	0.000
Left postcentral gyrus	22	3/4	−54	−24	39	3.28	0.001
Left hippocampus	36	31	−36	−30	−3	3.25	0.001
			−27	−36	0	2.96	0.002
Right putamen	30		33	0	−15	3.17	0.001
			33	−3	−6	3.01	0.001
			36	3	−24	2.6	0.005
Right inferior frontal gyrus	26	10/46/47	48	39	0	3.15	0.001
undefined	24		9	12	−33	3.13	0.001
			0	15	−33	2.87	0.002
Right anterior cerebellum lobe	62		3	−36	−27	3.05	0.001
			3	−57	−30	2.73	0.003
			3	−48	−30	2.65	0.004
Left medial frontal gyrus	19	6	−15	−27	39	2.87	0.002
			−3	−21	39	2.69	0.004
**BRAIN AREAS SHOWING NEGATIVE RELATIONSHIP BETWEEN WM AND ReHo**							
Bilateral posterior cerebellum lobe	1121		24	−60	−42	4.88	0.000
			−3	−78	−51	4.56	0.000
			−9	−78	−39	4.42	0.000
Right superior and middle frontal gyrus	30		21	54	9	4.03	0.000
Left superior and middle temporal gyrus	58	21/22/37/39	−60	−57	9	4	0.000
			−57	−63	0	3.28	0.001
Right precuneus	55	7	6	−60	57	3.85	0.000
			12	−72	60	2.76	0.003
Right parahippocampal gyrus	25	28/34	18	3	−24	3.44	0.000
			18	3	−15	2.89	0.002
Right medial frontal gyrus	36	11	6	39	−15	3.05	0.001

## Discussion

The present study was designed to examine *COMT* and *BDNF* gene interactions on WM and resting state fMRI (i.e., ReHo). As hypothesized, the *COMT* and *BDNF* had main and interaction effects on WM. The positive effect of the *COMT* Val allele on cognitive abilities is consistent with previous research with Asian samples (Qian et al., 2009; Yeh et al., 2009; Wang et al., 2016), but opposite of previous findings from Western samples that *COMT*-Met carriers showed better cognitive performance in both patients (Bilder et al., 2002; Joober et al., 2002) and healthy individuals (Caldú et al., 2007; Sheldrick et al., 2008). The main effect of *BDNF* on WM was modest, yielding no significant post hoc contrasts. Indeed, previous research has reported mixed findings, with some studies linking the Val allele to better performance on learning (Soliman et al., 2010), episodic memory (Egan et al., 2003), and WM tasks (Echeverria et al., 2005; Rybakowski et al., 2006), whereas others showed conflicting findings (Hansell et al., 2007; Schofield et al., 2009). Some of these conflicting results may well have been due to potential interactions between *BDNF* and *COMT* as found in our study on WM and ReHo, as well as in previous studies on paired-associates learning, stimulation-induced cortical plasticity (Witte et al., 2012), gray matter volumes (Mortby et al., 2014), and functional connectivity (Wang et al., 2015; Li et al.'s, 2016).

Most relevant to our work, Li et al.'s (2016) found that *COMT-M*+*/BDNF-M*+ and *COMT-VV/BDNF-VV* groups showed higher global functional connectivity density than the other two groups (*COMT-VV/BDNF-M*+ and *COMT-M*+*/BDNF-VV*). These results have been explained by interactive effects of *BDNF* and *COMT* on dopamine levels. Both *COMT* and *BDNF* influence the cytoarchitecture and connectivity among the subregions. Specifically, *BDNF* can affect the axonal and dendritic plasticity of neurons and their natural death (Ghosh et al., 1994), whereas *COMT* modulates the level of dopamine in the PFC (Okazawa et al., 1992; Nishio et al., 1998; Venero et al., 2000). In terms of their interaction, *BDNF* can stimulate dopamine activity (Blöchl and Sirrenberg, 1996; Murer et al., 2001). Previous biochemical research (Mannisto and Kaakkola, 1999; Winterer and Weinberger, 2004; Savitz et al., 2006; Peciña et al., 2014) showed that the *COMT* Met allele and the *BDNF* Val allele were linked to higher dopamine levels than their respective counterparts. Consequently the combination of *COMT-*M+ and *BDNF-*VV is expected to lead to a high level of dopamine, and the combination of *COMT-*VV and *BDNF-*M+ a low level of dopamine. In contrast, the other two combinations (*COMT-*VV and *BDNF-*VV; *COMT-*M+ and *BDNF-*M+) are expected to show moderate levels of dopamine. Li et al. (2016) finding that the latter two groups (with presumed medium levels of dopamine signaling) showed higher global functional connectivity density than the other two groups (either low or high levels of dopamine signaling) fits the commonly accepted inverted U-shaped association between dopamine level and outcomes (see Wang et al., 2015; Li et al.'s, 2016 for detailed discussions).

Mapping onto Li et al. (2016) findings, we found consistent U-shaped associations between presumed dopamine level and ReHo of five regions in the frontal lobe. These associations were statistically significant in terms of both interactions and *post-hoc* contrasts. As an index of a specific voxel's synchronization with its neighbors, ReHo reflects functional specificity of a brain region (Zang et al., 2004). Altered ReHo has been widely considered as an early indicator of brain dysfunction in patients (Wu et al., 2009, 2011; Chen et al., 2012; Cui et al., 2016). For example, a few studies have documented increased ReHo in the right dorsolateral PFC of patients with schizophrenia (Cui et al., 2016) and in the right medial frontal gyrus of patients with Parkinson's disease (Wu et al., 2009). Wu et al. (2009) further showed that when patients with Parkinson's disease were treated with levodopa, their right frontal gyrus showed decreased ReHo. Moreover, lower ReHo in a given region may indicate that it is more likely to be modulated by other regions (Yu et al., 2014), which may enhance the updating function in the WM process (Cools and Robbins, 2004).

Moreover, for the first time, the current study found the *COMT* and *BDNF* had interaction effect on ReHo. Moreover, ReHo in one of the regions (rSMFG) had a modest negative correlation with WM, which was confirmed by whole-brain analysis. The rSMFG has been consistently related to WM in previous lesion studies (Jacobsen and Nissen, 1937; Milner, 1963), electrophysiological studies (Fuster and Alexander, 1971; Funahashi et al., 1989, 1993), rTMS studies (Mottaghy et al., 2002), and fMRI studies (D'Esposito et al., 2000; Burgess and Braver, 2010; Marklund and Persson, 2012).

Consistent with the imaging data showing the importance of a moderate level of dopamine, individuals with the combination of *COMT-*VV and *BDNF-*VV (a combination expected to produce a medium amount of dopamine) showed top performance on the WM task. Surprisingly, however, the other combination (*COMT-*MM and *BDNF-*MM) that is also expected to produce a medium amount of dopamine showed low scores on the WM task. It should be noted, of course, that this group of minor allele homozygotes had the smallest sample size. A subsequent reanalysis of the data with Met carriers showed that *COMT-*M+*/BDNF-*M+ did not differ from *COMT*-M+/*BDNF*-VV and *COMT*-VV/*BDNF*-M+ (see Figure S1). Future research needs to include a larger sample in order to understand the group of *COMT-MM* and *BDNF-MM*.

Finally, although our results were generally consistent with other studies of the interaction effects of *COMT* and *BDNF* on other brain functions, our results need to be interpreted with caution due to several limitations of the present study. First, as mentioned earlier, our sample included too few *COMT* minor allele homozygotes to allow for a detailed analysis of the subjects. Second, the correlation between ReHo of rSMFG and WM was modest. It would not have survived a correction for multiple comparisons. Third, we did not collect any WM task-related fMRI data. Fourth, we did not administer a post-scan questionnaire to assess whether subjects followed the instructions not to fall asleep and not to think of anything in particular during scanning. Finally, given the ethnic differences in the effects of *COMT* on WM, it is imperative to directly compare these effects between Asian and non-Asian populations.

## Author contributions

Conceived and designed the experiments: Chuansheng C, QH, GX, WW, QD. Performed the experiments: GX, WW, YH. Analyzed the data: WC, MX. Wrote the paper: WC, Chunhui C, KW, and Chuansheng C.

## Funding

This work was supported by the National Natural Science Foundation of China (31571150), the 863 program (2015AA020912), the Fundamental Research Funds for the Central Universities (2012LYB05), the 111 Project (B07008) of the Ministry of Education of China. We thank all graduate research assistants who helped with data collection.

### Conflict of interest statement

The authors declare that the research was conducted in the absence of any commercial or financial relationships that could be construed as a potential conflict of interest. The reviewer KR and handling Editor declared their shared affiliation, and the handling Editor states that the process nevertheless met the standards of a fair and objective review.
