# Supplementary material for: Interaction Effects of BDNF and COMT Genes on Resting-State Brain Activity and Working Memory
Source: Front Hum Neurosci. 2016 Nov 2;10:540. doi: 10.3389/fnhum.2016.00540 (PMC5091010; doi:10.3389/fnhum.2016.00540)
Supplement: Supplementary file 1 [file DataSheet1.DOCX]

**Interaction effects of *BDNF* and *COMT* genes on resting-state brain activity and working memory**

***Supplemental Materials***

**Table S1.** Main effects of *COMT* on ReHo with age, gender, and IQ as covariates

| **Brain areas** | **Cluster size (Voxels)** | **Brodmann Areas (BA)** | | **Peak coordinates in MNI** | | | **T value** |
| --- | --- | --- | --- | --- | --- | --- | --- |
|  |  |  |  | **x** | **y** | **z** |  |
| ***Brain areas where Val homozygotes had higher ReHo than Met carriers*** | | | | | | | |
| Right fusiform gyrus | 40 | 20/37 | 33 | | -36 | -21 | 3.77 |
|  |  |  | 33 | | -39 | -12 | 3.44 |
| Left precentral gyrus | 25 | 3/4 | -24 | | -21 | 57 | 3.68 |
| Left cerebellum posterior lobe | 25 |  | -30 | | -66 | -27 | 3.59 |
|  |  |  | -39 | | -60 | -30 | 2.50 |
| Left putamen | 20 |  | -24 | | -15 | 3 | 3.53 |
|  |  |  | -27 | | -9 | -6 | 2.41 |
| Bilateral cerebellum anterior lobe | 94 |  | 12 | | -33 | -24 | 3.28 |
|  |  |  | -3 | | -30 | -33 | 2.87 |
|  |  |  | -12 | | -36 | -15 | 2.86 |
| Right hippocampus | 43 |  | 42 | | -24 | -15 | 3.27 |
|  |  |  | 33 | | -15 | -15 | 3.09 |
|  |  |  | 33 | | -24 | -9 | 3.07 |
| Right fusiform gyrus | 19 | 19 | 18 | | -87 | -21 | 3.24 |
| Right parahippocampa gyrus | 25 | 27/30 | 3 | | -45 | -3 | 3.24 |
|  |  |  | 9 | | -39 | -3 | 3.07 |
|  |  |  | 9 | | -39 | -12 | 2.98 |
| Right middle occipital gyrus | 18 | 19/39 | 39 | | -72 | 21 | 2.68 |
| ***Brain areas where Val homozygotes had lower ReHo than Met carriers*** | | | | | | | |
| Left superior temporal gyrus | 50 | 13/22/38 | -51 | | 6 | -6 | 5.05 |
|  |  |  | -39 | | 6 | -12 | 2.67 |
| Right inferior frontal gyrus | 23 | 47 | 27 | | 24 | -6 | 4.36 |
|  |  |  | 30 | | 33 | 0 | 2.53 |
| Bilateral precentral gyrus | 30 | 4/22/42/43 | 66 | | -6 | 12 | 3.70 |
| Right precentral gyrus | 42 | 6 | 39 | | -15 | 42 | 3.48 |
|  |  |  | 42 | | -18 | 24 | 2.65 |
|  |  |  | 36 | | -18 | 30 | 2.59 |
| Left superior temporal gyrus | 30 | 9 | -12 | | 51 | 21 | 3.23 |
|  |  |  | -9 | | 60 | 36 | 2.55 |
| Left middle frontal gyrus | 27 |  | -39 | | 45 | -9 | 3.21 |
|  |  |  | -33 | | 42 | 0 | 2.83 |
|  | 22 | 7/40 | -33 | | -57 | 48 | 3.13 |
| Right superior temporal gyrus | 17 | 41 | 45 | | -39 | 6 | 3.10 |
| Left superior frontal gyrus | 21 | 10/11 | -21 | | 48 | -12 | 2.92 |
|  |  |  | -27 | | 57 | -3 | 2.74 |
|  |  |  | -12 | | 51 | -9 | 2.44 |
| Right postcentral gyrus | 17 | 2/3/5 | 21 | | -42 | 69 | 2.83 |
|  |  |  | 24 | | -33 | 69 | 2.56 |

**Table S2.** Main effects of *BDNF* on ReHo with age, gender, and IQ as covariates

| **Brain areas** | **Cluster size (Voxels)** | **Brodmann Areas (BA)** | **Peak coordinates in MNI** | | | **T value** |
| --- | --- | --- | --- | --- | --- | --- |
|  |  |  | **x** | **y** | **z** |  |
| ***Brain areas where Val homozygotes had higher ReHo than Met carriers*** | | | | | | |
| Right lingual gyrus | 190 | 17/18/19/23/30 | 18 | -78 | -15 | 3.96 |
|  |  |  | 27 | -69 | -12 | 3.59 |
|  |  |  | 18 | -78 | 9 | 3.40 |
| Left lingual gyrus | 219 | 17/18 | -9 | -66 | -6 | 3.61 |
|  |  |  | -18 | -78 | -6 | 3.31 |
|  |  |  | -15 | -84 | -12 | 3.28 |
| Right lingual gyrus and limbic lobe | 108 | 18/19/29/30/36/37 | 9 | -54 | 3 | 3.57 |
|  |  |  | 24 | -45 | -9 | 3.52 |
|  |  |  | 27 | -48 | 3 | 3.33 |
| Right postcentral gyrus | 103 | 1/2/3/4/6 | 54 | -15 | 45 | 3.56 |
|  |  |  | 48 | -24 | 51 | 3.53 |
|  |  |  | 54 | -12 | 33 | 3.05 |
| Right postcentral gyrus | 47 | 2/3/5/40 | 33 | -39 | 63 | 3.53 |
|  |  |  | 39 | -30 | 63 | 2.40 |
| Precentral and postcentral gyrus | 47 | 3/4/6 | -60 | -15 | 33 | 3.51 |
| Left middle occipital gyrus | 71 | 17/18 | -12 | -96 | 3 | 3.50 |
|  |  |  | -21 | -99 | 0 | 3.02 |
|  |  |  | -18 | -90 | 12 | 2.90 |
| Right precuneus | 39 | 7/19 | 21 | -78 | 42 | 3.33 |
|  |  |  | 24 | -75 | 33 | 2.95 |
| Right superior frontal gyrus | 21 | 9/10 | 33 | 51 | 27 | 3.19 |
| Left anterior cingulate | 22 | 24/32 | -6 | 21 | 27 | 2.98 |
| Left lingual gyrus | 39 | 19/30 | -21 | -51 | 0 | 2.96 |
|  |  |  | -24 | -51 | -18 | 2.87 |
|  |  |  | -15 | -48 | -9 | 2.81 |
| ***Brain areas where Val homozygotes had lower ReHo than Met carriers*** | | | | | | |
| Left inferior temporal gyrus | 31 | 20 | -51 | -36 | -27 | 4.34 |
| Left parahippocampal gyrus | 83 | 20/28/35/36 | -27 | -18 | -27 | 3.95 |
|  |  |  | -24 | -24 | -18 | 3.03 |
| Left inferior frontal gyrus | 59 | 11/38/47 | -36 | 18 | -27 | 3.62 |
|  |  |  | -15 | 33 | -24 | 2.90 |
|  |  |  | -24 | 24 | -24 | 2.83 |
| Bilateral brainstem | 120 |  | 0 | -33 | -51 | 3.55 |
|  |  |  | -3 | -27 | -42 | 3.50 |
|  |  |  | 3 | -15 | -33 | 3.29 |
| Left cerebellum posterior lobe | 57 |  | -30 | -48 | -48 | 3.50 |
|  |  |  | -30 | -60 | -48 | 3.09 |
| Right superior and medial frontal gyrus | 25 | 8/9 | 18 | 27 | 33 | 3.49 |
|  |  |  | 27 | 21 | 39 | 2.99 |
| Right midbrain | 25 |  | 3 | -30 | -3 | 3.44 |
|  |  |  | 3 | -36 | -12 | 2.68 |
| Right parahippocampal gyrus | 46 | 20/28/35/36 | 21 | -9 | -33 | 3.40 |
|  |  |  | 24 | -15 | -27 | 3.36 |
|  |  |  | 36 | -18 | -27 | 2.88 |
| Left inferior frontal gyrus | 38 | 28/47 | -27 | 9 | -15 | 3.38 |
|  |  |  | -18 | 12 | -15 | 2.80 |
| Left paracentral lobule | 34 | 5/7 | -6 | -42 | 54 | 3.18 |
|  |  |  | -18 | -42 | 51 | 3.18 |
| Left middle temporal gyrus | 19 | 21 | -42 | 3 | -36 | 3.16 |
|  |  |  | -42 | 9 | -42 | 2.83 |
| Frontal lobe | 17 |  | 33 | 30 | 15 | 3.06 |
| Left cerebellum posterior lobe | 17 |  | -33 | -81 | -54 | 2.95 |
| Frontal lobe | 23 |  | 27 | 9 | 21 | 2.93 |
|  |  |  | 39 | 12 | 21 | 2.92 |
| Rectal gyrus | 19 | 11 | 6 | 18 | -24 | 2.86 |
|  |  |  | -3 | 9 | -24 | 2.68 |
| Right cerebellum posterior lobe | 29 |  | 39 | -78 | -51 | 2.78 |
|  |  |  | 24 | -81 | -54 | 2.69 |
| Right cerebellum posterior lobe | 28 |  | 33 | -54 | -57 | 2.67 |
|  |  |  | 24 | -51 | -54 | 2.61 |

**Table S3**. Brain areas showing significant COMT and BDNF gene interaction effects on ReHo with age, gender and IQ as covariates (without global signal regression during the preprocessing).

| **Brain areas** | **Cluster size (Voxels)** | **Brodmann Areas (BA)** | **Peak coordinates in MNI** | | | **T value** |
| --- | --- | --- | --- | --- | --- | --- |
|  |  |  | **x** | **y** | **z** |  |
| Left superior frontal gyrus | 39 | 10 | -9 | 54 | 12 | 3.17 |
|  |  |  | -15 | 48 | 24 | 3.15 |
| left middle frontal gyrus | 30 | 47 | -30 | 42 | 0 | 3.44 |
|  |  |  | -39 | 45 | -9 | 2.67 |
| Left medial frontal gyrus | 25 | 6 | 0 | -18 | 66 | 3.04 |
|  |  |  | -3 | -27 | 66 | 2.9 |
| Right superior and medial frontal gyrus | 24 | 9/10 | 6 | 51 | 21 | 3.23 |
|  | 17 |  | -27 | 39 | 27 | 3.38 |
| Right inferior orbitofrontal gyrus | 17 | 38/47 | 36 | 24 | -24 | 3.22 |
| Right parahippocampal Gyrus | 53 | 28/35/46 | 12 | -33 | -15 | 3.64 |
|  |  |  | 18 | -24 | -24 | 3.34 |
| Right sub-gyral | 37 |  | 24 | 27 | 9 | 3.32 |
|  |  |  | 27 | 33 | 0 | 3.07 |
|  |  |  | 18 | 33 | 6 | 2.76 |
| Left sub-gyral | 36 |  | -27 | -27 | 24 | 3.78 |
| Left thalamus | 34 |  | -6 | -6 | -3 | 3.65 |
|  |  |  | -9 | -3 | 6 | 2.96 |
|  |  |  | 21 | -27 | -12 | 3.27 |
| Left superior temporal gyrus | 32 | 41 | -42 | -33 | 3 | 4.03 |
| Left lingual gyrus | 29 | 18 | -33 | -72 | -6 | 3.91 |
|  |  |  | -21 | -75 | -3 | 2.79 |
| Right sub-gyral | 25 |  | 30 | 15 | 27 | 3.27 |
| undefined | 25 |  | -6 | -27 | -57 | 3.15 |
|  |  |  | 3 | -27 | -57 | 3.07 |
| Left fusiform gyrus | 21 | 37 | -45 | -45 | -15 | 3.76 |
| left brainstem | 19 |  | -3 | -27 | -9 | 3.29 |
| Left cuneus | 19 |  | -12 | -84 | 21 | 3.25 |
| Right superior temporal gyrus | 19 | 39 | 45 | -54 | 18 | 3.52 |
| Right inferior parietal lobule | 19 | 40 | 69 | -33 | 33 | 3.3 |

**Table S4.** Brain areas showing significant correlations between WM and ReHo throughout the whole brain, with AlphaSim correction (p < 0.01) (without global signal regression during the preprocessing).

| **Brain areas** | **Cluster size (Voxels)** | **Brodmann Areas (BA)** | **Peak coordinates in MNI** | | | **T value** |
| --- | --- | --- | --- | --- | --- | --- |
|  |  |  | **x** | **y** | **z** |  |
| ***Brain areas showing positive relationship between WM and ReHo*** | | | | | | |
| Right insula | 344 | 6/13/44 | 24 | -24 | 0 | 3.72 |
|  |  |  | 33 | -3 | 6 | 3.67 |
|  |  |  | 36 | -3 | -6 | 3.65 |
| Left insula | 158 | 40/41/42/43/44 | -33 | -15 | 18 | 3.29 |
|  |  |  | -60 | -18 | 15 | 3.21 |
|  |  |  | -39 | 3 | 6 | 3.16 |
| Right anterior cerebellum | 95 |  | -6 | -39 | -18 | 3.33 |
|  |  |  | 3 | -54 | 0 | 3.22 |
|  |  |  | 9 | -48 | -9 | 3.2 |
| Left middle cingulate gyrus | 80 | 24/32 | 12 | 6 | 33 | 3.59 |
|  |  |  | 0 | 6 | 45 | 3.11 |
|  |  |  | -9 | 0 | 45 | 2.94 |
| Right cingulate gyrus | 57 |  | 6 | 9 | 15 | 3.75 |
|  |  |  | 9 | 0 | 21 | 3.02 |
|  |  |  | 15 | -9 | 33 | 3 |
| Left postcentral gyrus | 38 | 2/3 | -54 | -24 | 39 | 3.07 |
|  |  |  | -54 | -18 | 30 | 3.07 |
| Left hippocampus | 32 |  | -33 | -30 | -3 | 3.1 |
|  |  |  | -27 | -21 | -6 | 2.93 |
|  |  |  | -27 | -36 | 0 | 2.92 |
| Left middle temporal gyrus | 32 |  | -51 | -48 | -6 | 3.82 |
| Left precuneus | 31 |  | -15 | -51 | 21 | 3.19 |
|  |  |  | -6 | -42 | 12 | 2.87 |
| Left middle frontal gyrus | 29 | 6 | -18 | -12 | 60 | 3.42 |
| Right superior frontal gyrus | 29 | 6 | 27 | -9 | 54 | 3.24 |
| Left superior occipital gyrus | 26 | 7/18/19 | -12 | -84 | 24 | 4.27 |
| Left inferior parietal lobule | 23 | 40 | -48 | -45 | 36 | 3.4 |
|  |  |  | -54 | -39 | 45 | 2.48 |
| Left superior frontal gyrus | 23 | 11 | -27 | 45 | -15 | 3.23 |
| Right inferior orbitofrontal gyrus | 22 | 47 | 45 | 24 | -15 | 3.13 |
| Right precentral gyrus | 20 | 4/6 | 63 | -12 | 39 | 2.9 |
|  |  |  | 57 | -12 | 30 | 2.48 |
| Left medial frontal gyrus | 21 | 6 | -6 | 0 | 60 | 3.5 |
| Left posterior cerebellum | 19 |  | -12 | -69 | -27 | 3.43 |
| Left postcentral gyrus | 19 | 3/4 | -18 | -36 | 60 | 2.96 |
|  |  |  | -30 | -33 | 66 | 2.82 |
|  |  |  | -21 | -30 | 69 | 2.63 |
| ***Brain areas showing negative relationship between WM and ReHo*** | | | | | | |
| Bilateral posterior cerebellum | 1164 |  | 27 | -60 | -42 | 4.87 |
|  |  |  | -3 | -78 | -51 | 4.55 |
|  |  |  | -9 | -81 | -39 | 4.25 |
| Right medial frontal gyrus | 58 | 11 | 6 | 39 | -15 | 3.37 |
|  |  |  | 0 | 51 | -15 | 2.75 |
| Left superior temporal gyrus | 57 | 28/34/38 | -33 | 18 | -30 | 3.84 |
|  |  |  | -15 | 6 | -30 | 3 |
|  |  |  | -18 | 3 | -21 | 2.91 |
| Right parahippocampal gyrus | 50 |  | 18 | 3 | -24 | 3.79 |
|  |  |  | 15 | 0 | -15 | 3.44 |
|  |  |  | 24 | 3 | -30 | 3.11 |
| Right Precuneus | 48 |  | 6 | -60 | 57 | 3.42 |
|  |  |  | 12 | -75 | 54 | 2.8 |
|  |  |  | 9 | -69 | 63 | 2.6 |
| Left superior and middle temporal gyrus | 35 | 21/22/39 | -60 | -57 | 9 | 3.63 |
| Right superior and medial gyrus | 34 | 8/9 | 21 | 54 | 39 | 2.89 |
|  |  |  | 24 | 39 | 48 | 2.68 |
|  |  |  | 12 | 45 | 45 | 2.66 |
| Right superior and medial frontal gyrus | 30 | 10 | 21 | 51 | 9 | 3.49 |
| Right sub-gyral | 29 |  | 24 | 21 | -3 | 3.55 |
|  |  |  | 24 | 33 | 3 | 3.48 |
| Right precuneus | 27 |  | 6 | -57 | 33 | 3.27 |
|  |  |  | 6 | -54 | 24 | 2.82 |
| Left cerebellum | 25 | 28/34 | -48 | -48 | -42 | 3.82 |
|  |  |  | -42 | -42 | -45 | 3.46 |
| Right superior occipital gyrus | 24 |  | 39 | -90 | 18 | 3.18 |
|  |  |  | 45 | -81 | 18 | 2.82 |
|  |  |  | 36 | -87 | 30 | 2.73 |
| Right superior frontal gyrus | 21 | 10 | 24 | 66 | 18 | 3.38 |
| Right inferior temporal gyrus | 18 |  | 45 | 6 | -45 | 3.09 |
| Right cerebellum | 17 |  | 24 | -39 | -45 | 3.1 |
|  |  |  | 18 | -45 | -48 | 2.76 |

**Figure S1.** Interaction effect of *COMT* and *BDNF* in the 417 sample (2×2 genotypes ANCOVA). Post hoc LSD test showed that *COMT*-VV was higher than *COMT*-M+ for *BDNF*-VV, p < .01)


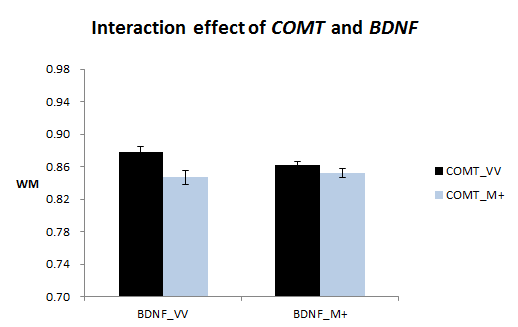


**Figure S2**. Effects of COMT and BDNF on mean ReHo of right superior and medial frontal gyrus (rSMFG).
